# Intra-Individual Comparison of 18F-PSMA-1007 and 18F-FDG PET/CT in the Evaluation of Patients With Prostate Cancer

**DOI:** 10.3389/fonc.2020.585213

**Published:** 2021-02-01

**Authors:** Xing Zhou, YingChun Li, Xiao Jiang, XiaoXiong Wang, ShiRong Chen, TaiPeng Shen, JinHui You, Hao Lu, Hong Liao, Zeng Li, ZhuZhong Cheng

**Affiliations:** ^1^Radiation Oncology Key Laboratory of Sichuan Province, PET/CT Centre, Sichuan Cancer Hospital, Chengdu, China; ^2^Department of Nuclear Medicine, Affiliated Hospital of North Sichuan Medical College, Nanchong, China; ^3^Department of Nuclear Medicine & Radiotherapy, Air Force Hospital of Western Theater Command, Chengdu, China; ^4^Radiation Oncology Key Laboratory of Sichuan Province, Department of Urology, Sichuan Cancer Hospital, Chengdu, China

**Keywords:** 18F-PSMA-1007, 18F-FDG, PET/CT, prostate cancer, pitfalls

## Abstract

**Purpose:**

18F labelled PSMA-1007 presents promising results in detecting prostate cancer (PC), while some pitfalls exists meanwhile. An intra-individual comparison of 18F-FDG and 18F-PSMA-1007 in patients with prostate cancer were aimed to be performed in the present study. Then, the pitfalls of 18F-PSMA-1007 PET/CT in imaging of patients with prostate cancer were analyzed.

**Methods and Material:**

21 prostate cancer patients underwent 18F-PSMA-1007 PET/CT as well as 18F-FDG PET/CT before treatment. All positive lesions were noticed in both 18F-PSMA-1007 PET/CT and 18F-FDG PET/CT, then differentiated PC metastasis from benign lesions. the SUVmax, SUVmean and TBR of lesions, up to 10 metastases and 10 benign lesions per patients were recorded (5 for bone, 5 for soft tissue metastasis ). The distribution of positive lesions were analyzed for two imaging. Detection rates, SUVmax, SUVmean and TBR in 18F-PSMA-1007 PET/CT and 18F-FDG PET/CT were compared, respectively. The optimal cut-off values of SUVmax, SUVmean for metastases vs. benign lesions was found through areas under ROC in 18F-PSMA-1007.

**Results:**

The detection rates of primary lesions in 18F-PSMA-1007 PET/CT was higher than that of 18F-FDG PET/CT(100% (21/21) vs. 67%(14/21)). For extra- prostatic lesions, 18F-PSMA-1007 PET/CT revealed 124 positive lesions, 49(49/124, 40%) attributed to a benign origin; 18F-FDG PET/CT revealed 68 positive lesions, 14(14/68, 21%) attributed to a benign origin. The SUVmax, SUVmean, TBR of primary tumor in 18F-PSMA-1007 PET/CT was higher than that in 18F-FDG PET/CT (15.20 vs. 4.20 for SUVmax; 8.70 vs. 2.80 for SUVmean; 24.92 vs. 4.82 for TBR, respectively); The SUVmax, SUVmean, TBR of metastases in 18F-PSMA-1007 PET/CT was higher than that in 18F-FDG PET/CT (10.72 vs. 4.42 for SUVmax; 6.67 vs. 2.59 for SUVmean; The TBR of metastases was 13.3 vs. 7.91). For 18F-FDG PET/CT, the SUVmax, SUVmean in metastases was higher than that in benign lesions (4.42 vs. 3.04 for SUVmax, 2.59 vs. 1.75 for SUVmean, respectively). Similarly, for 18F-PSMA-1007 PET/CT, the SUVmax, SUVmean in metastases was significantly higher than that in benign lesions(10.72 vs. 3.14 for SUVmax, 6.67 vs. 1.91 for SUVmean, respectively), ROC suggested that SUVmax=7.71, SUVmean=5.35 might be the optimal cut-off values for metastases vs. benign lesions.

**Conclusion:**

The pilot study suggested that 18F-PSMA-1007 showed superiority over 18F-FDG because its high detecting rate of PC lesions and excellent tumor uptake. While non-tumor uptake in 18F-PSMA-1007 may lead to misdiagnosis, recognizing these pitfalls and careful analysis can improve the accuracy of diagnosis.

## Introduction

Prostate cancer is the second most common cancer in men (1). Early detection and accurate staging leads to improved clinical decision making. Different from traditional imaging (e.g., computed tomography (CT), magnetic resonance imaging (MRI)), whole-body imaging seems to be an advantage for PET/CT. Recently, the study of prostate-specific membrane antigen (PSMA) is growing and suggesting impressive results in the diagnosis and staging of prostate cancer (2–4).

18F labelled prostate-specific membrane antigen (PSMA)-1007 is a novel PSMA-based radiopharmaceutical, it was introduced into clinical practice because its excellent tumor uptake and high sensitivity for detecting lesions (5–7). Furthermore, 18F-PSMA-1007 is mainly cleared by hepatobiliary system, providing clinical practice benefits (8–10). 18F-FDG is the most widely used radiotracer, which is effective for diagnosis and staging of prostate cancer (11). So far, 18F-PSMA-1007 has not been compared with 18F-FDG yet.

Recently, with the extensive application of PSMA-target tracer, the pitfalls of PSMA-target PET has been found increasingly (12–15). The uptake of PSMA-ligand in other malignant and benign pathologies (e.g., celiac and other ganglia, fracture, degenerative changes) causes challenge to clinical diagnosis. Recognizing these limitations can be essential.

The aim of present study was to perform an intra-individual comparison of 18F-FDG and 18F-PSMA-1007 in the evaluation of patients with prostate cancer. Then analyzed the pitfalls that may appear when conducting 18F-PSMA-1007 PET/CT in order to reduce the probability of misdiagnosis.

## Materials and Methods

### Patients

A total of 21 patients (median age, 66 y; range, 50-82 y) with pathologically diagnosed as prostate cancer underwent 18F-PSMA-1007 PET/CT and 18F-FDG PET/CT before treatment. 18 (86%) of these patients were diagnosed as prostate cancer with perineal prostate biopsy; two (9%) patients were confirmed by biopsy of pelvic lymph node, ala of ilium, respectively; one (5%) patient was diagnosed by biopsy with cystoscope. The Gleason score was available for 16 patients, the median Gleason score was 9 (range 7–10). The treatment of patients was as follows, eight (38%) of the patients received exclusively androgen deprivation therapy (ADT), two (10%) patients was received ADT after docetaxel chemotherapy. Four (19%) patients was treated with only radical prostatectomy. Seven (33%) was treated with ADT after radical prostatectomy.

The study was ethically approved by the Institutional Ethics Committee (Ethics Committee of Sichuan Cancer Hospital, JS-2017-01-02) and in accordance to the local regulations of China. All patients signed a written informed consent form. The patients characteristics were listed in **Table 1**.

**Table 1:** Patient characteristics.

PatientNo.	Age(y)	Gleason score	Days from PSMA PET/CT To FDG PET/CT	initial PSA (ng/mL)	Local tumor growth (n)		Lymph node metastases (n)		bone metastases (n)	
					FDG	PSMA	FDG	PSMA	FDG	PSMA
1	73		4	183.00	1	1	>10	>10	>10	>10
2	63	4+3	9	5.00	1	1	0	1	0	3
3	71	4+5	1		1	2	0	0	0	0
4	74	3+4	4	10.65	0	1	0	1	0	0
5	72	4+4	7	30.14	1	1	1	5	0	2
6	82		3	100.00	1	1	2	3	>10	>10
7	64	5+4	3	153.00	0	2	>10	>10	>10	>10
8	80		1	70.50	0	>2	1	5	2	6
9	63	4+3	5	147.00	1	1	0	5	1	1
10	68	5+5	34	16.22	1	2	0	0	0	1
11	68	4+3		9.90	1	>2	0	7	0	3
12	74	4+4	18	25.63	1	>2	0	0	0	1
13	50		7	15.50	1	1	0	3	1	2
14	65	4+5	9		0	1	0	4	0	0
15	66	4+4	6	184.00	1	1	1	5	0	0
16	66	5+4	5	50.80	0	1	4	7	0	0
17	56	3+4	9	31.60	0	1	0	2	2	4
18	65	4+5	7	91.30	1	2	5	6	>10	>10
19	75		7		1	1	0	0	0	2
20	56	5+5	11	200.00	1	>2	0	2	0	0
21	64	4+4	3	20.01	0	1	0	0	6	6

### Radiosynthesis and Quality Control

18F-PSMA-1007 was synthesized by a one-step method using an automated radiosynthesizer (Sumitomo, Japan) as was described (16). 18F^-^ was acquired by (18F)/H_2_18O nuclear reaction, and then loaded onto quarternary methyla-minecolumn (Waters, America), After eluted by 0.75 ml tetrabutylammonium hydrogen carbonate (TBAHCO_3_) solution (ABX, Radeberg, Germany), it was transfered into reactor and followed by the addition of 0.4 ml anhydrous acetonitrile (Sigma, America), then removal of water with the temperature of 95°C. 1.2 ml dimethyl sulfoxide (ABX, Radeberg, Germany) which dissolved with PSMA-1007 precursor (ABX, Radeberg, Germany) was added into reactor and performed fluorination reaction at 85°C for 10 min. Then diluted with 6 ml of 5% ethano and loaded onto PS-H+ and C18ec (ABX, Radeberg, Germany) followed by 4 ml of 30% ethanol. Final product was eluted with 4 ml of 30% ethanol and was added into 0.1 ml of 100 mg/L Vitamin C solution, 36 mL of 0.9% NaCl, then was sterilized by 0.22 μm filter (Millipore, America). High-performance liquid chromatography (HPLC, Shimadzu, Japan) was performed to test chemical purity, Further quality control (appearance, color, clarity, PH, and adionuclidic purity) was done and in compliance with current pharmacopoeias. The synthesis of 18F-FDG was performed as reported by Gallagher et al. (17).

### Imaging Procedures

To reduce the mutual interference of the two radiotracers, imaging was carried out at different days. The median 6.5 (range 1.0–34.0) days passed from 18F-PSMA-1007 to 18F-FDG. Patients fasted for at least 6 h prior to injection of the 18F-FDG and blood sugar level is lower than 15 mg/L. The injected activity of 18F-FDG were mean 388 ± 55 MBq (range 281–503 MBq) and scanning was performed 60 min after injection, while the injected activity of 18F-PSMA-1007 were 348 ± 52 MBq (range 266–458 MBq), and according to Giesel et al. (5) imaging began 180 min after injection. All scans were obtained on a Biograph mCT-64 PET/CT scanner (Siemens). Non-enhanced low-dose (1.3–1.5 mSv) CT scan was performed with CT parameters (140 keV, 42 mA) section width of 8 mm, pitch of 0.8, and CT datas were used for attenuation correction. The PET-scan, PET was acquired in 3-D FlowMotion with an acquisition time of 2 min per bed position. Both scans was performed from vertex to the mid-thigh. Images were reconstructed with an ordered-subset expectation-maximization iterative reconstruction algorithm (three iterations, 21 subsets).

### Image Analysis and Quantification

All images were evaluated by two double board-certified nuclear medicine physician. Volumes of interest (VOI) were drawn around lesions using an maximum standardized uptake value (SUVmax) threshold of isocontour of 42% (18). Intra-prostatic lesions were defined as positive if the tracer-uptake was focal and higher than surrounding prostate tissue (19). Other soft tissue and bone metastases were judged as positive when there were obvious morphological changes meanwhile corresponding lesions showed increased radiotracer-uptake above normal surroundings (20). Benign lesions were recognised based on typical pitfalls (e.g., ganglia, fracture, degenerative changes, and unspecific lymph nodes) in PSMA ligand PET imaging and information from CT (14). All PET positive lesions were counted and lesions grouped into: (a) local tumor growth, (b) soft tissue metastases [including lymph node (LN) metastases, other soft tissue metastases (e.g., lung, liver)] (c) bone metastases, (d) benign lesions. In accordance with previous studies, obturator muscle was chosen as background and VOI was drawn aroud it (19, 21). Tumor-to-background ratio (TBR) were defined as SUVmax of lesions/SUVmax of obturator muscle. for the SUVs (SUVmax and SUVmean), TBR of primary tumor, up to 10 metastases per patients were recorded (five for bone, five for soft tissue metastasis); the SUVs (SUVmax and SUVmean) of up to 10 benign lesions per patients were recorded.

### Statistical Analysis

Statistical analysis was performed using SPSS software, version 24.0 (IBM Corp.). The nonparametric Mann-Whitney U test for two independent samples was used to compare the SUVs, TBR of all lesions. When performed 18F-PSMA-1007 PET/CT, Areas under receiver operating characteristic curves (ROC) were calculated and optimal cut-off values of SUV in metastases vs. benign lesions were calculated using the Youden’s index. P < 0.05 were considered significant.

## Results

The median initial PSA was 41.20 ng/ml (range, 5.00–200.00 ng/ml). The median of 6.5 d (range, 1.0–34.0 d) passed from 18F-PSMA-1007 PET/CT to 18F-FDG PET/CT. The SUVmax of urinary bladder was significantly lower in 18F-PSMA-1007 PET/CT than in 18F-FDG PET/CT (median SUVmax of 2.40 (range 0.60–11.00) vs. 15.64 (range 6.19-35.47), P < 0.001). No statistically significant difference was found when evaluating the SUVmax of 18F-FDG PET/CT and 18F-PSMA-1007 PET/CT for obturator muscle (median SUVmax of 0.75 vs. 0.70, P = 0.061) (**Table 2**).

**Table 2:** Comparison of mean SUV and TBR of lesions in 18F-PSMA-1007 PET and 18F-FDG PET.

	18F-PSMA-1007				18F-FDG			
	n	SUVmax	SUVmean	TBR	n	SUVmax	SUVmean	TBR
urinary bladder	21	2.40 (0.60–11.00)			21	15.64(6.19–35.47)		
obturator muscle	21	0.70(0.35–0.92)			21	0.75(0.57–1.64)		
local lesion	21	15.20 (6.20–75.00)	8.70(3.80–43.00)	24.92(8.41–117.06)	14	4.20(2.80–10.50)	2.80(1.60–6.30)	4.82(1.00–14.00)
metastase	75	10.72 (1.42–79.70)	6.67(0.89–50.16)	13.3(1.61–96.02)	54	4.42(1.05–12.41)	2.59(0.86–7.81)	7.91(1.28–96.02)
benign lesions	49	3.14(1.26–12.98)	1.91(0.89–8.30)		14	3.04(1.71–5.60)	1.75(0.86–3.38)	

TBR, tumor-to-background ratio.

### Local Lesion Finding and Uptake

**Figure 1:**
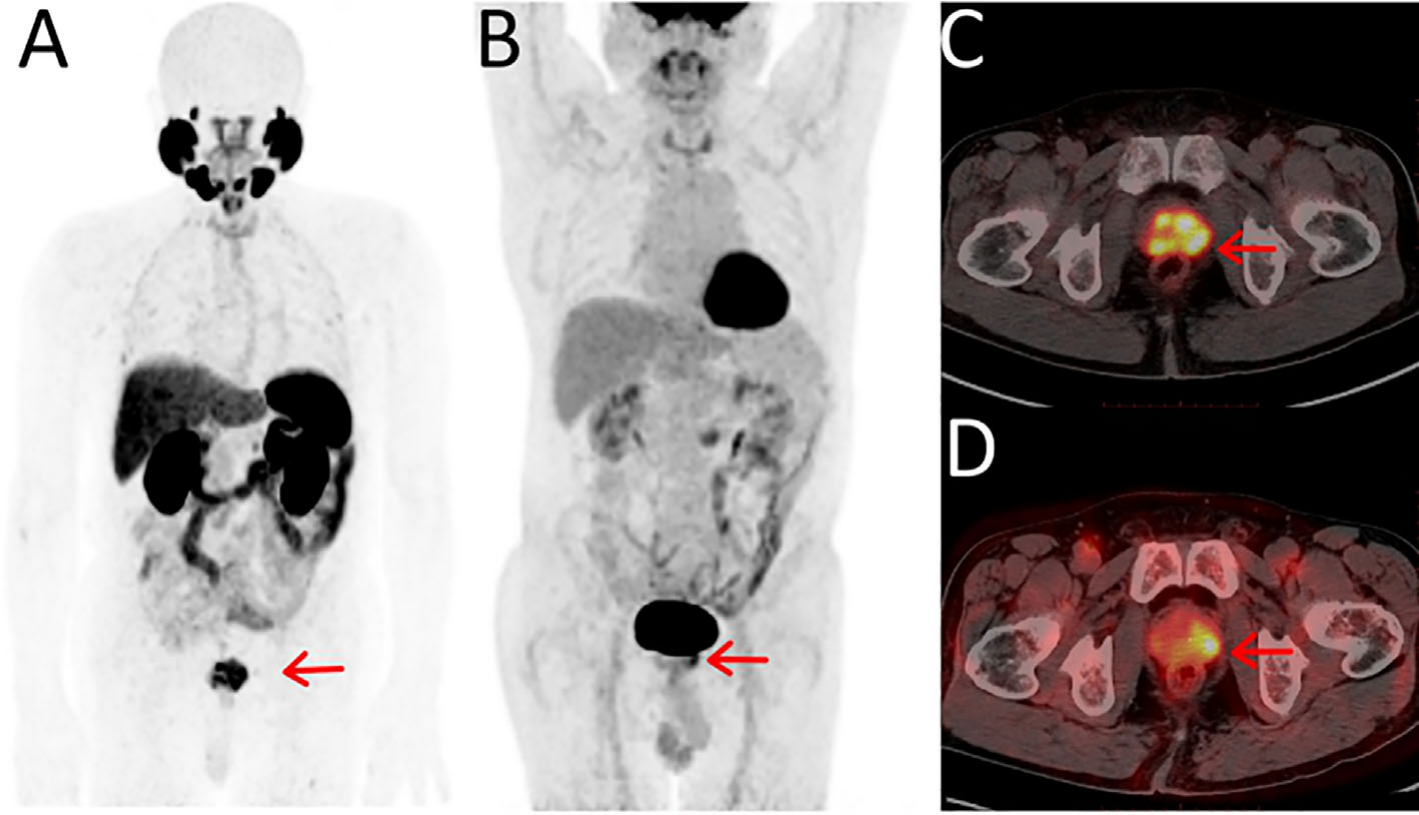
Maximum-intensity projections of PET examinations using ^18^F-PSMA-1007 **(A)** and ^18^F-FDG **(B)**. Axial PET/CT for ^18^F-PSMA-1007 **(C)** and ^18^F-FDG **(D)**. The 74-y-old patient presented with PSA serum level of 42.5 ng/ml at time of examinations. With positive biopsy (Gleason score 7 [4 + 3]) and was treatment-na¨ıve at the time of the examinations. **(C)** ^18^F-PSMA-1007 PET/CT showed PSMA-positive lesions in the prostate and the lesions had obvious multifocality, the SUVmax was 15.18. **(D)** ^18^F-FDG PET/CT showed a focal positive lesions in the prostate and the SUVmax was 5.88.

Among these 21 prostate cancer patients, 18F-PSMA-1007 PET/CT detected all patients (100%), eight (38%) cases of them had obvious multifocality. 14 of 21 cases (67%) was identified by 18F-FDG PET/CT, and none of them was found multifocality (**Table 1**, **Figure 1**). SUVmax, SUVmean of local lesions was significantly higher in 18F-PSMA-1007 PET/CT than in 18F-FDG PET/CT (median SUVmax of 15.20 (range 6.20–75.00) vs. 4.20 (range 2.80–10.50), P < 0.001), (median SUVmean of 8.70 (range 3.80–43.00) vs. 2.80 (range 1.60–.30), P < 0.001). TBR of local lesions was significantly higher in 18F-PSMA-1007 PET/CT than in 18F-FDG PET/CT (median TBR of 24.92 (range 8.41–117.06) vs. 4.82 (range 1.00–14.00), P < 0.001) (**Table 2**).

### Metastases Finding and Uptake

**Figure 2:**
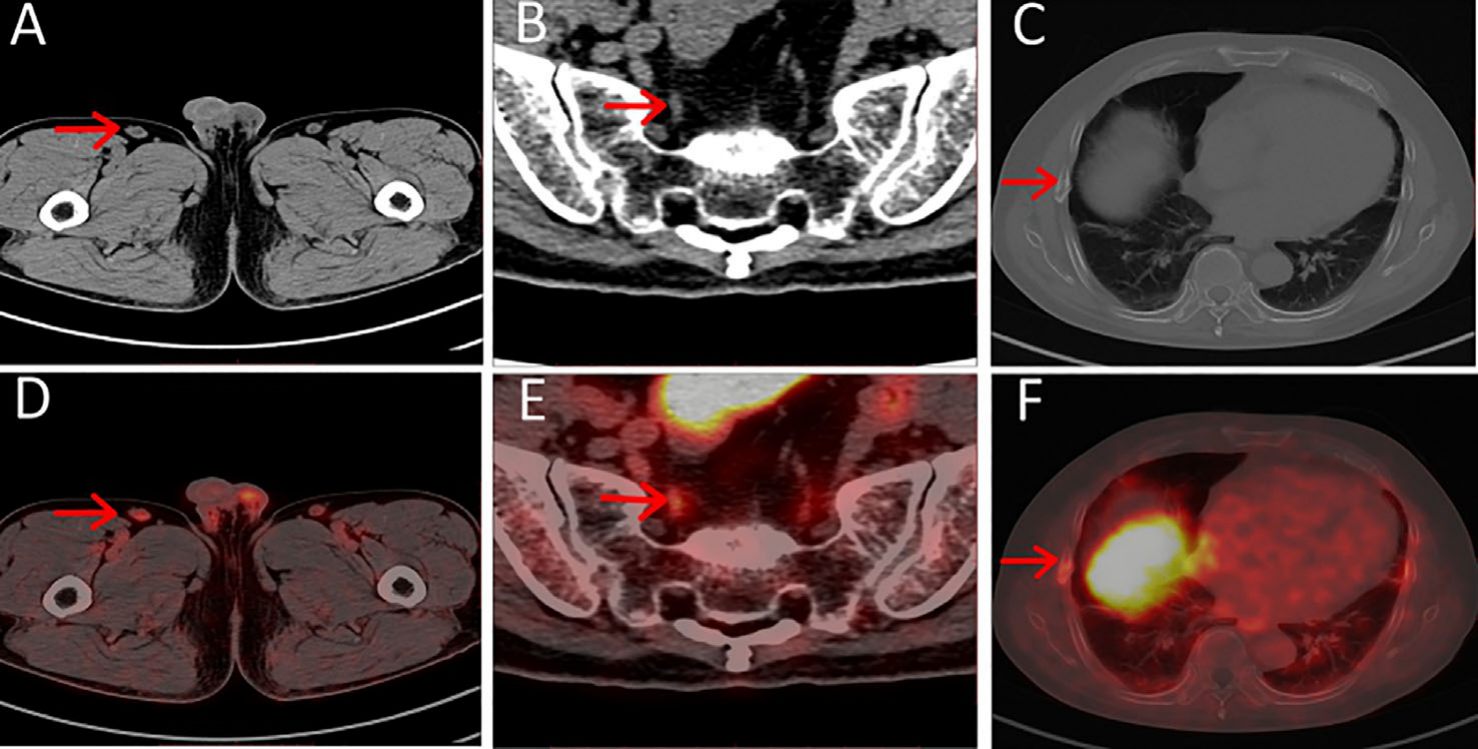
**(A–C)** Axial CT, **(D–F)** axial PET/CT of pitfalls in ^18^F-PSMA-1007. Unspecific PSMA-ligand uptake in right inguinal lymph node **(A, D)**, PSMA-ligand uptake in pelvic ganglia **(B, E)** and PSMA-ligand uptake in right non-displaced rib fracture **(C, F)**.

**Figure 3:**
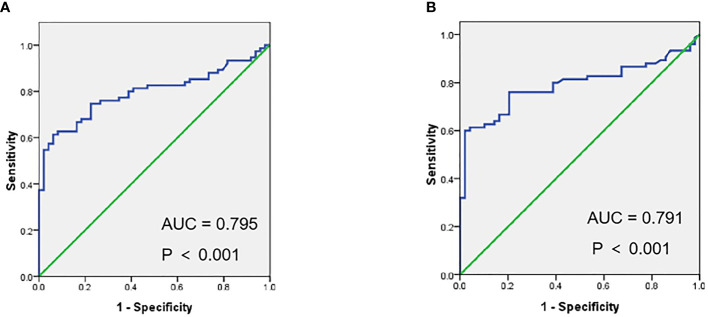
Receiver operating characteristic curves (ROC) of SUVmax **(A)**, SUVmean **(B)** in metastases vs. benign lesions for 18F-PSMA-1007 PET/CT.

**Figure 4:**
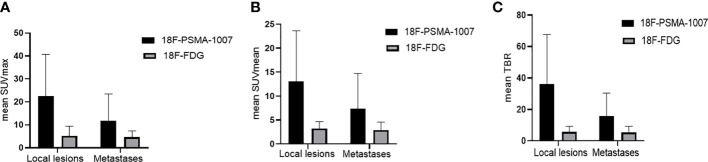
**(A)** Comparison of mean SUVmax and its SD of ^18^F-PSMA-1007 and ^18^F-FDG for local lesions and metastases. **(B)** Comparison of mean SUVmean and its SD of ^18^F-PSMA-1007 and ^18^F-FDG for local lesions and metastases. **(C)** Comparison of mean TBR (tumor-to-background ratio) and its SD of ^18^F-PSMA-1007 and ^18^F-FDG for local lesions and metastases.

18F-PSMA-1007 PET/CT found 124 lesions with focal PSMA-ligand uptake, 49 (40%) of them attributed to benign origin (17 (35%) lesions suspicious of ganglia, 12 (24%) lesions attributed to unspecific lymph nodes, five (10%) lesions for fracture, five (10%) lesions attributed to degenerative changes, three (6%) lesions for unspecific soft tissue, seven (15%) lesions showed focal increased radiotracer-uptake above normal surroundings in PSMA PET without corresponding morphological changes in CT (two lesions for bone, five for others); 75(60%) attributed to metastases [50(67%) lesions for bone metastases (**Table 3**), 25(33%) for lymph node metastases, no other soft tissue metastases was found]. For details see **Figure 2**. The SUVmax, SUVmean for suspicious metastases was significantly higher than probably benign (median SUVmax of 10.72 (range 1.42–79.70) vs. 3.14 (range 1.26–12.98), P < 0.001), (median SUVmean of 6.67 (range 0.89–50.16) vs. 1.91 (range 0.89–8.30), P < 0.001). (**Table 2**, **Figure 4**). ROC showed that optimal cut-off values of SUV in metastases vs. benign lesions was SUVmax = 7.71, (Areas under curve (AUC) = 0.795, P < 0.001), SUVmean = 5.35 (AUC = 0.791, P < 0.001) (**Figure 3**).

**Table 3:** The distribution of PSMA-positive lesions and FDG-positive lesions.

Distribution	18F-PSMA-1007	18F-FDG
All	124	68
**metastase**	75 (60%)	54 (79%)
Bone metastase	50	32
Soft tissue metastase	25	22
**benign lesions**	49 (40%)	14 (21%)
ganglia	17	
unspecific lymph nodes	12	7
fracture	5	3
degenerative changes	5	2
unspecific soft tissue	3	2
increasly PSMA-ligand uptake without correlate on CT images	7	

18F-FDG PET/CT detected a total of 68 FDG-positive lesions, 14 (21%) with benign origin [seven (50%) lesions attributed to unspecific lymph nodes, three (22%) lesions for fracture, two (14%) for degenerative changes, two (14%) for others], 54 (79%) with metastases [32 (59%) lesions attributed to bone metastases, 22 (41%) lesions suspicious of LN metastases]. For details see **Table 3**. The SUVmax, SUVmean for suspicious metastases was significantly higher than probably benign [median SUVmax of 4.42 (range 1.05–12.41) vs. 3.04 (range 1.71–5.60), P = 0.036), (median SUVmean of 2.59 (range 0.86–7.81) vs. 1.75 (range 0.86–3.38), P = 0.014] (**Table 2**).

No statistically significant was found when comparing the SUVs of lesions attributed to benign with 18F-PSMA-1007 and 18F-FDG (median SUVmax 3.14 vs. 3.04, P > 0.05), SUVmean (median SUVmean 1.91 vs. 1.75, P > 0.05). SUVmax, SUVmean of metastases was significantly higher for 18F-PSMA-1007 PET/CT than for 18F-FDG PET/CT (median SUVmax of 10.72 (range 1.42–79.70) vs. 4.42 (range 1.05–12.41), P < 0.001), (median SUVmean of 6.67 (range 0.89–50.16) vs. 2.59 (range 0.86–7.81), P < 0.001). TBR of metastases was significantly higher in 18F-PSMA-1007 PET/CT than in 18F-FDG PET/CT (median TBR of 13.3 (range 1.61–96.02) vs. 7.91(range 1.28–96.02), P < 0.001 (**Figure 4**).

## Discussion

This retrospective study mainly focused on comparing the detection rate of local lesions and metastases (both LN and bone metastases) in 18F-FDG PET/CT and 18F-PSMA-1007 PET/CT. Simultaneously, performing a comparison of lesions attributed to benign origin and lesions attributed to metastases in 18F-FDG PET/CT and 18F-PSMA-1007 PET/CT, respectively.

Detection rate for local lesions in 18F-PSMA-1007 PET/CT was higher than in 18F-FDG PET/CT (100% (21/21) for 18F-PSMA-1007 PET/CT, 67% (14/21) for 18F-FDG PET/CT) and 18F-PSMA-1007 PET/CT was more likely to find lesions with multifocality (eight cases for 18F-PSMA-1007 PET/CT, none for 18F-FDG PET/CT). This might be explained by the following reasons: Firstly, PSMA is a type II transmembrane glycoprotein that is strongly overexpressed in PCa cells (both primary tumor and metastases) and low in benign prostate tissue (22–24), making 18F-PSMA-1007 PET/CT a promising technique for detecting and locating prostate cancer. Furthermore, hepatobiliary elimination seems to be another advantage for 18F-PSMA-1007, while 18F-FDG mainly excreted *via* urinary tract (17, 25); low bladder/ureter activity in 18F-PSMA-1007 PET/CT make it possible to differentiate primary tumor and pelvic lymph node metastases from the bladder urinalysis activity (26). In present study, we found 18F-PSMA-1007 PET/CT was more likely to detect metastases (both LN and bone metastases) than 18F-FDG PET/CT (75 lesions for 18F-PSMA-1007 PET/CT, 54 for 18F-FDG PET/CT). However, a recently published study shows no significant difference was found when comparing the detection rate in 18F-PSMA-1007 and 18F-DCFPyL (27). Both tracers belong to the same family of PSMA ligands and labelled by the same radioisotope (18F) may explain the phenomenon (28).

Consistent with previous studies, we found that 18F-PSMA-1007 PET/CT shows high tumor-to-background ratios (TBR) in lesions with prostate cancer (6), median TBR of 24.92 in local lesions with prostate cancer, median TBR of 13.30 in lymph node metastases and bone metastases with prostate cancer. The median TBR of 18F-FDG PET/CT in local lesions with prostate cancer, lymph node metastases, and bone metastases with prostate cancer was 4.82 and 7.91, respectively. After injection of 18F-PSMA-1007 for 3 h, the uptake of radio-tracer in prostate cancer lesions demonstrated a remarkable increasing and leading to the improvement of tumor-to-background ratios (5), it makes tumor lesions more visible in 18F-PSMA-1007 PET/CT than in 18F-FDG PET/CT.

Recent studies suggest that PSMA-target PET shows some pitfalls in clinical application, especially in 18F labeled PSMA, as it may be expressed in other malignant and benign pathologies, even some normal tissues (6, 13, 14, 29) and these findings are in line with ours. We found that some PSMA-positive lesions attributed to benign origin (e.g., benign lymph nodes, ganglia, and skeletal fracture) and the reason of this phenomenon is unclear yet. To our knowledge, salivary glands, liver, gallbladder, etc. non-prostate tissues show uptake in 18F-PSMA-1007 PET (5, 7), providing the possibility of PSMA-uptaking in benign lesions. Moreover, PSMA present both in peri-tumoral capillaries and inflammatory-associated neovasculature may explain the uptake of benign lesions (14). The arising of these pitfalls leads to an increasing in false positive and brings us challenges. Differentiating suspicious metastases from these potential diagnostic pitfalls may be of increased importance.

In present study, we found that the uptake of PSMA ligand tracer in probable Pca metastases was significantly higher than in benign lesions (10.72 vs. 3.14 for SUV max, 6.67 vs. 1.91 for SUVmean), which was consistent with previous studies (15). And ROC shows that SUVmax ≥7.71 was more likely to be Pca metastases (AUC = 0.795, P < 0.001) than SUVmax <7.71, SUVmean ≥5.35 was more likely to be Pca metastases (AUC = 0.791, P < 0.001) than SUVmax <5.35. These findings make it possible to differentiate suspicious metastases and benign lesions. Furthermore, lesions attributed to benign origin can be identified by CT (with 80% in benign lesions, 96% in coeliac ganglia) (14, 30), due to their typical shapes and locations. More importantly, the clinical medical records (e.g., other imaging data, history of fracture, inflammation) providing essential information when evaluating benign lesions.

In accordance with previous study (13, 14), we found that the most prevalent pitfall in 18F-PSMA-1007 PET/CT was non-specific radiotracer uptake in ganglia, with 17 (35%) lesions attributed to ganglia (including cervical, coeliac, or sacral ganglia). A recent study publication by Krohn et al. demonstrated that up to 94.0% of prostate cancer patients with PSMA-PET/CT show intense PSMA-ligand uptake in at least one coeliac ganglia (15). In current study, the distribution of radio-tracer uptake in other benign lesion with 18F-PSMA-1007 PET/CT was as follows, unspecific lymph nodes, fracture, degenerative changes, unspecific soft tissues, focal increasingly PSMA-ligand uptake showing no clear correlate on CT images, which were along with the previous study (12–14). Recently, a study found that a high number of PSMA-ligand uptake in the ribs without corresponding morphological changes in CT (14), which was different from our finding [only two (28%) lesions]. Small population was involved in present study may be the reason leading to this difference.

Lacking histopathology verification of the PSMA-positive lesions is the major limitation in present study. However, the uptake of lesions, CT images, and clinical medical records provide the possibility to identify benign lesions. Additionally, small patient population is another limitation, larger comparison trials will be needed in future studies.

## Conclusion

The study demonstrated that 18F-PSMA-1007 showed superiority in detecting Pca lesions (both primay and metastases) than 18F-FDG and the uptaking in benign lesions was more likely to be found in 18F-PSMA-1007. Emphasizing the known of pitfalls, evaluating PET and CT images as well as clinical medical records make it available to avoid a misdiagnosis in 18F-PSMA-1007 PET/CT.

## Data Availability Statement

The datasets presented in this article are not readily available. Requests to access the datasets should be directed to zxing94@126.com.

## Ethics Statement

The study was ethically approved by Sichuan Cancer Hospital Ethics Committee and in accordance to the local regulations of China. All patients signed a written informed consent form.

## Author Contributions

XZ, YL, XJ, XW, and ZZ designed the project, XZ and YL wrote the manuscript. HLu, HLi, and ZL organized data. SC and TS analyzed data. JY and ZC reviewed the data and the manuscript. All authors contributed to the article and approved the submitted version.

## Funding

This study was supplied by the Sichuan Science and Technology program (2019YJ0574; 2020YFS0421) and the Health research project of Sichuan Province (20PJ117).

## Conflict of Interest

The authors declare that the research was conducted in the absence of any commercial or financial relationships that could be construed as a potential conflict of interest.

## References

[1] Bray F Ferlay J Soerjomataram I Siegel RL Torre LA Jemal A Global cancer statistics 2018: GLOBOCAN estimates of incidence and mortality worldwide for 36 cancers in 185 countries. CA: Cancer J Clin (2018) 68(6):394–424. 10.3322/caac.21492 30207593

[2] Perera M Papa N Christidis D Wetherell D Hofman MS Murphy DG Sensitivity, Specificity, and Predictors of Positive (68)Ga-Prostate-specific Membrane Antigen Positron Emission Tomography in Advanced Prostate Cancer: A Systematic Review and Meta-analysis. Eur Urol (2016) 70(6):926–37. 10.1016/j.eururo.2016.06.021 27363387

[3] Afshar-Oromieh A Babich JW Kratochwil C Giesel FL Eisenhut M Kopka K The Rise of PSMA Ligands for Diagnosis and Therapy of Prostate Cancer. J Nucl Med (2016) 57(Suppl 3):79s–89s. 10.2967/jnumed.115.170720 27694178

[4] Sterzing F Kratochwil C Fiedler H Katayama S Habl G Kopka K (68)Ga-PSMA-11 PET/CT: a new technique with high potential for the radiotherapeutic management of prostate cancer patients. Eur J Nucl Med Mol Imaging (2016) 43(1):34–41. 10.1007/s00259-015-3188-1 26404016 PMC4771815

[5] Giesel FL Hadaschik B Cardinale J Radtke J Vinsensia M Lehnert W F-18 labelled PSMA-1007: biodistribution, radiation dosimetry and histopathological validation of tumor lesions in prostate cancer patients. Eur J Nucl Med Mol Imaging (2017) 44(4):678–88. 10.1007/s00259-016-3573-4 PMC5323462 27889802

[6] Cardinale J Schäfer M Benešová M Bauder-Wüst U Leotta K Eder M Preclinical Evaluation of (18)F-PSMA-1007, a New Prostate-Specific Membrane Antigen Ligand for Prostate Cancer Imaging. J Nucl Med (2017) 58(3):425–31. 10.2967/jnumed.116.181768 27789722

[7] Rahbar K Afshar-Oromieh A Bögemann M Wagner S Schäfers M Stegger L (18)F-PSMA-1007 PET/CT at 60 and 120 minutes in patients with prostate cancer: biodistribution, tumour detection and activity kinetics. Eur J Nucl Med Mol Imaging (2018) 45(8):1329–34. 10.1007/s00259-018-3989-0 29541812

[8] Giesel FL Kesch C Yun M Cardinale J Haberkorn U Kopka K 18F-PSMA-1007 PET/CT Detects Micrometastases in a Patient With Biochemically Recurrent Prostate Cancer. Clin Genitourinary Cancer (2017) 15(3):e497–e9. 10.1016/j.clgc.2016.12.029 28131751

[9] Giesel FL Will L Kesch C Freitag M Kremer C Merkle J Biochemical Recurrence of Prostate Cancer: Initial Results with [(18)F]PSMA-1007 PET/CT. J Nucl Med (2018) 59(4):632–5. 10.2967/jnumed.117.196329 29419475

[10] Giesel FL Knorr K Spohn F Will L Maurer T Flechsig P Detection Efficacy of (18)F-PSMA-1007 PET/CT in 251 Patients with Biochemical Recurrence of Prostate Cancer After Radical Prostatectomy. J Nucl Med (2019) 60(3):362–8. 10.2967/jnumed.118.212233 PMC6424235 30042163

[11] Jadvar H Prostate cancer: PET with 18F-FDG, 18F- or 11C-acetate, and 18F- or 11C-choline. J Nucl Med (2011) 52(1):81–9. 10.2967/jnumed.110.077941 PMC3012154 21149473

[12] Keidar Z Gill R Goshen E Israel O Davidson T Morgulis M 68Ga-PSMA PET/CT in prostate cancer patients - patterns of disease, benign findings and pitfalls. Cancer Imaging (2018) 18(1):39. 10.1186/s40644-018-0175-3 30382889 PMC6211573

[13] Sheikhbahaei S Afshar-Oromieh A Eiber M Solnes LB Javadi MS Ross AE Pearls and pitfalls in clinical interpretation of prostate-specific membrane antigen (PSMA)-targeted PET imaging. Eur J Nucl Med Mol Imaging (2017) 44(12):2117–36. 10.1007/s00259-017-3780-7 28765998

[14] Rauscher I Krönke M König M Gafita A Maurer T Horn T Matched-Pair Comparison of (68)Ga-PSMA-11 PET/CT and (18)F-PSMA-1007 PET/CT: Frequency of Pitfalls and Detection Efficacy in Biochemical Recurrence After Radical Prostatectomy. J Nucl Med (2020) 61(1):51–7. 10.2967/jnumed.119.229187 PMC6954457 31253741

[15] Krohn T Verburg FA Pufe T Neuhuber W Vogg A Heinzel A [(68)Ga]PSMA-HBED uptake mimicking lymph node metastasis in coeliac ganglia: an important pitfall in clinical practice. Eur J Nucl Med Mol Imaging (2015) 42(2):210–4. 10.1007/s00259-014-2915-3 25248644

[16] Zhou X Shen TP Yao YT Lu H Chen SR Xiao DQ Synthesis of 18F-PSMA-1007 by one-step method and PET/CT imaging in prostate cancer. Chin J Nucl Med Mol Imaging (2019) 39(10):606–9. 10.3760/cma.j.issn.2095-2848.2019.10.007

[17] Gallagher BM Ansari A Atkins H Casella V Christman DR Fowler JS Radiopharmaceuticals XXVII. 18F-labeled 2-deoxy-2-fluoro-d-glucose as a radiopharmaceutical for measuring regional myocardial glucose metabolism in vivo: tissue distribution and imaging studies in animals. J Nucl Med (1977) 18(10):990–6. 903484

[18] Miccò M Vargas HA Burger IA Kollmeier MA Goldman DA Park KJ Combined pre-treatment MRI and 18F-FDG PET/CT parameters as prognostic biomarkers in patients with cervical cancer. Eur J Radiol (2014) 83(7):1169–76. 10.1016/j.ejrad.2014.03.024 24767630

[19] Uprimny C Kroiss AS Decristoforo C Fritz J von Guggenberg E Kendler D (68)Ga-PSMA-11 PET/CT in primary staging of prostate cancer: PSA and Gleason score predict the intensity of tracer accumulation in the primary tumour. Eur J Nucl Med Mol Imaging (2017) 44(6):941–9. 10.1007/s00259-017-3631-6 28138747

[20] Eiber M Maurer T Souvatzoglou M Beer AJ Ruffani A Haller B Evaluation of Hybrid ^6 8^ Ga-PSMA Ligand PET/CT in 248 Patients with Biochemical Recurrence After Radical Prostatectomy. J Nucl Med (2015) 56(5):668–74. 10.2967/jnumed.115.154153 25791990

[21] Fendler WP Schmidt DF Wenter V Thierfelder KM Zach C Stief C 68Ga-PSMA PET/CT Detects the Location and Extent of Primary Prostate Cancer. J Nucl Med (2016) 57(11):1720–5. 10.2967/jnumed.116.172627 27261520

[22] Demirci E Sahin OE Ocak M Akovali B Nematyazar J Kabasakal L Normal distribution pattern and physiological variants of 68Ga-PSMA-11 PET/CT imaging. Nucl Med Commun (2016) 37(11):1169–79. 10.1097/mnm.0000000000000566 27333090

[23] Mease RC Foss CA Pomper MG PET imaging in prostate cancer: focus on prostate-specific membrane antigen. Curr Top Med Chem (2013) 13(8):951–62. 10.2174/1568026611313080008 PMC4067736 23590171

[24] Afshar-Oromieh A Malcher A Eder M Eisenhut M Linhart HG Hadaschik BA PET imaging with a [68Ga]gallium-labelled PSMA ligand for the diagnosis of prostate cancer: biodistribution in humans and first evaluation of tumour lesions. Eur J Nucl Med Mol Imaging (2013) 40(4):486–95. 10.1007/s00259-012-2298-2 23179945

[25] Rahbar K Weckesser M Ahmadzadehfar H Schäfers M Stegger L Bögemann M Advantage of (18)F-PSMA-1007 over (68)Ga-PSMA-11 PET imaging for differentiation of local recurrence vs. urinary tracer excretion. Eur J Nucl Med Mol Imaging (2018) 45(6):1076–7. 10.1007/s00259-018-3952-0 29445927

[26] Kuten J Fahoum I Savin Z Shamni O Gitstein G Hershkovitz D Head-to-Head Comparison of (68)Ga-PSMA-11 with (18)F-PSMA-1007 PET/CT in Staging Prostate Cancer Using Histopathology and Immunohistochemical Analysis as a Reference Standard. J Nucl Med (2020) 61(4):527–32. 10.2967/jnumed.119.234187 31562225

[27] Giesel FL Will L Lawal I Lengana T Kratochwil C Vorster M Intraindividual Comparison of (18)F-PSMA-1007 and (18)F-DCFPyL PET/CT in the Prospective Evaluation of Patients with Newly Diagnosed Prostate Carcinoma: A Pilot Study. J Nucl Med (2018) 59(7):1076–80. 10.2967/jnumed.117.204669 29269569

[28] Kopka K Benešová M Bařinka C Haberkorn U Babich J Glu-Ureido-Based Inhibitors of Prostate-Specific Membrane Antigen: Lessons Learned During the Development of a Novel Class of Low-Molecular-Weight Theranostic Radiotracers. J Nucl Med (2017) 58(Suppl 2):17s–26s. 10.2967/jnumed.116.186775 28864607

[29] Jochumsen MR Dias AH Bouchelouche K Benign Traumatic Rib Fracture: A Potential Pitfall on 68Ga-Prostate-Specific Membrane Antigen PET/CT for Prostate Cancer. Clin Nucl Med (2018) 43(1):38–40. 10.1097/rlu.0000000000001871 29076907

[30] Wang ZJ Webb EM Westphalen AC Coakley FV Yeh BM Multi-detector row computed tomographic appearance of celiac ganglia. J Comput Assist Tomogr (2010) 34(3):343–7. 10.1097/RCT.0b013e3181d26ddd 20498533

